# Metabolic-Associated Fatty Liver Disease: The Influence of Oxidative Stress, Inflammation, Mitochondrial Dysfunctions, and the Role of Polyphenols

**DOI:** 10.3390/ph17101354

**Published:** 2024-10-10

**Authors:** Raissa Bulaty Tauil, Paula Takano Golono, Enzo Pereira de Lima, Ricardo de Alvares Goulart, Elen Landgraf Guiguer, Marcelo Dib Bechara, Claudia C. T. Nicolau, José Luiz Yanaguizawa Junior, Adriana M. R. Fiorini, Nahum Méndez-Sánchez, Ludovico Abenavoli, Rosa Direito, Vitor Engrácia Valente, Lucas Fornari Laurindo, Sandra Maria Barbalho

**Affiliations:** 1 Department of Biochemistry and Pharmacology, School of Medicine, Universidade de Marília (UNIMAR), Marília 17525-902, São Paulo, Brazil; 2 Postgraduate Program in Structural and Functional Interactions in Rehabilitation, School of Medicine, Universidade de Marília (UNIMAR), Marília 17525-902, São Paulo, Brazil; 3 Department of Biochemistry and Nutrition, School of Food and Technology of Marília (FATEC), Marília 17500-000, São Paulo, Brazil; 4 Liver Research Unit, Medica Sur Clinic & Foundation, Mexico City 14050, Mexico; nmendez@medicasur.org.mx; 5 Faculty of Medicine, National Autonomous University of Mexico, Mexico City 04510, Mexico; 6 Department of Health Sciences, University “Magna Graecia”, Viale Europa, 88100 Catanzaro, Italy; l.abenavoli@unicz.it; 7 Laboratory of Systems Integration Pharmacology, Clinical and Regulatory Science, Research Institute for Medicines, Universidade de Lisboa (iMed.ULisboa), Av. Prof. Gama Pinto, 1649-003 Lisbon, Portugal; rdireito@ff.ulisboa.pt; 8 Autonomic Nervous System Center, School of Philosophy and Sciences, São Paulo State University, Marília 17525-902, São Paulo, Brazil; 9 Department of Biochemistry and Pharmacology, School of Medicine, Faculdade de Medicina de Marília (FAMEMA), Marília 17519-030, São Paulo, Brazil; lucasffffor@gmail.com; 10 Research Coordination, UNIMAR Charity Hospital, Universidade de Marília (UNIMAR), Marília 17525-902, São Paulo, Brazil

**Keywords:** metabolic-associated fatty liver disease, MAFLD, liver disease, inflammation, oxidative stress, mitochondrial dysfunction, polyphenols

## Abstract

Metabolic-Associated Fatty Liver Disease (MAFLD) is a clinical–pathological scenario that occurs due to the accumulation of triglycerides in hepatocytes which is considered a significant cause of liver conditions and contributes to an increased risk of death worldwide. Even though the possible causes of MAFLD can involve the interaction of genetics, hormones, and nutrition, lifestyle (diet and sedentary lifestyle) is the most influential factor in developing this condition. Polyphenols comprise many natural chemical compounds that can be helpful in managing metabolic diseases. Therefore, the aim of this review was to investigate the impact of oxidative stress, inflammation, mitochondrial dysfunction, and the role of polyphenols in managing MAFLD. Some polyphenols can reverse part of the liver damage related to inflammation, oxidative stress, or mitochondrial dysfunction, and among them are anthocyanin, baicalin, catechin, curcumin, chlorogenic acid, didymin, epigallocatechin-3-gallate, luteolin, mangiferin, puerarin, punicalagin, resveratrol, and silymarin. These compounds have actions in reducing plasma liver enzymes, body mass index, waist circumference, adipose visceral indices, lipids, glycated hemoglobin, insulin resistance, and the HOMA index. They also reduce nuclear factor-KB (NF-KB), interleukin (IL)-1β, IL-6, tumor necrosis factor-α (TNF-α), blood pressure, liver fat content, steatosis index, and fibrosis. On the other hand, they can improve HDL-c, adiponectin levels, and fibrogenesis markers. These results show that polyphenols are promising in the prevention and treatment of MAFLD.

## 1. Introduction

Metabolic-Associated Fatty Liver Disease (MAFLD) is a clinical–pathological scenario that occurs due to the accumulation of triglycerides in hepatocytes which is considered a significant cause of liver conditions and contributes to an increased risk of death worldwide [1,2,3]. It has different stages, starting with a simple accumulation of triglycerides (non-alcoholic steatohepatitis—NASH), which can progress to inflammation and later to fibrosis, cirrhosis, or hepatocarcinoma. The pathogenesis of MAFLD has not been fully understood. Still, there is evidence that insulin resistance (IR) and associated subclinical inflammation, obesity, and metabolic syndrome are recognized origins in the course of this condition [4,5] and affect up to 30% of the world’s population [6,7,8].

Recently, the term non-alcoholic fatty liver disease (NAFLD) was changed to MAFLD, since this one better identifies patients at a higher risk of liver fibrosis and progression of the condition [9]. Moreover, other researchers proposed the term Metabolic Dysfunction-associated Steatotic Liver Disease (MASLD) to include at least one of five cardiometabolic risk factors. However, some authors investigated MAFLD and MASLD as predictors of an augmented risk of atherosclerotic cardiovascular disease. The authors included more than six thousand people that participated in the National Health and Nutrition Examination Survey cohort. Their results showed that MAFLD and MASLD were related to different risks for atherosclerotic cardiovascular disease. Notwithstanding, MAFLD predicted the risk of this condition more than MASLD [9,10,11,12].

**Figure 1:**
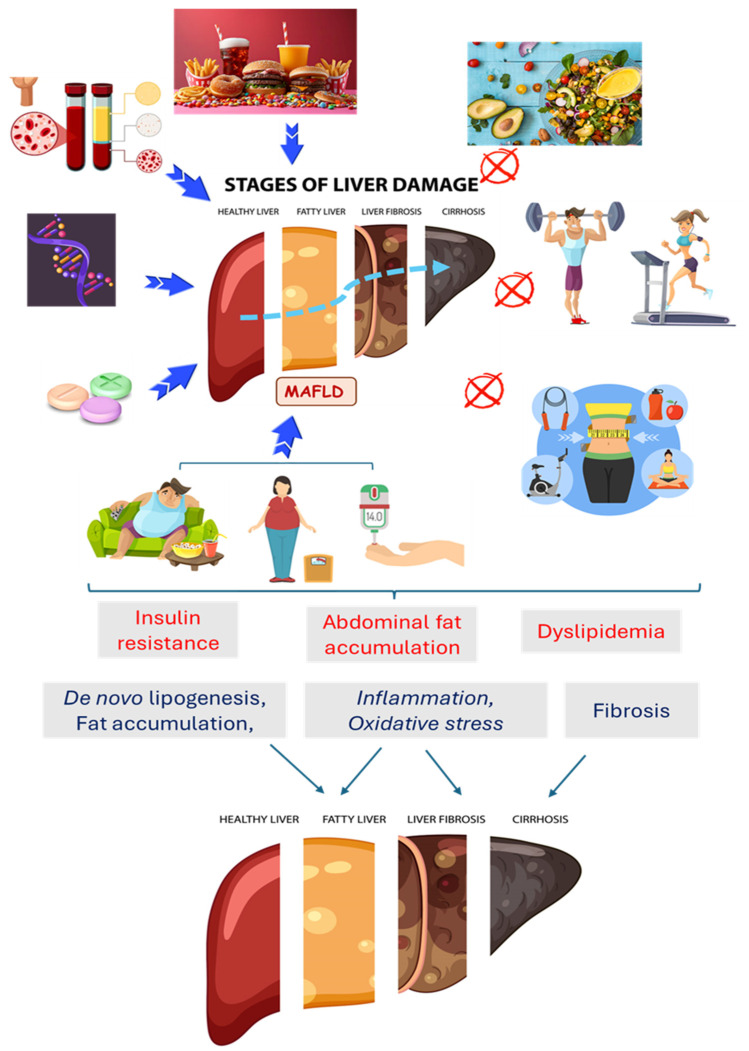
Factors related to the occurrence of Metabolic-Associated Fatty Liver Disease (MAFLD) and the possibility of the inhibition of this condition. An unhealthy diet, sedentary lifestyle, obesity, insulin resistance/diabetes, dyslipidemia, genetics, and excessive drug consumption are related to the pathogenesis of MAFLD and its progression to fibrosis, cirrhosis, and cancer. A healthy diet, physical exercise, and weight loss can improve metabolic conditions and can prevent or reduce MAFLD.

MAFLD is normally directly related to dyslipidemia, metabolic syndrome, obesity, and diabetes [13]. This condition causes the dysregulation of the brain–intestine–liver axis, and as a result, people with MAFLD tend to have greater cardiovascular risks and more severe fatty liver disease [14,15,16]. Even though the possible causes of MAFLD can involve the interaction of genetics, hormones, and nutrition, lifestyle (diet and sedentary lifestyle) is the most influential factor in developing this condition. Furthermore, if body weight decreases from 7% to 10%, MAFLD can be reversed in adults and children [17,18,19,20]. Figure 1 shows some aspects of MAFLD pathogenesis.

Diet can profoundly influence metabolic diseases. Food diets rich in fats, sugar, and ultra-processed foods are related to inflammatory and oxidative processes. A diet abundant in fruit and vegetables can reduce risk factors such as dyslipidemia, hyperglycemia, hypertension, obesity, inflammation, and oxidative stress [5,21,22,23,24,25,26,27], which are essential in the development and progression of MAFLD [28,29,30,31]. Phytocompounds comprise many natural chemical compounds that are beneficial to counter metabolic diseases [32,33,34,35,36,37,38,39,40,41,42]. Polyphenols are part of this group, and more than a thousand have been identified. Significant components of this class are phenols, polyphenols, carotenoids, phytosterols, isoprenoids, saponins, and dietary fibers [43].

Some polyphenols can reverse part of the liver damage related to inflammation, oxidative stress, or mitochondrial dysfunction, acting directly on the functioning, synthesis, and degradation of mitochondria and optimizing the functions of these cellular organelles. Among these polyphenols are anthocyanin, baicalin, catechin, curcumin, chlorogenic acid, didymin, epigallocatechin-3-gallate, luteolin, mangiferin, puerarin, punicalagin, resveratrol, and silymarin [44,45,46,47,48,49,50,51,52,53,54,55]. The incidence of liver disorders and associated conditions such as overweight/obesity, diabetes, and metabolic syndrome has grown exponentially. For these reasons, more research must be conducted to propose ways to mitigate risk factors for these conditions [56]. Therefore, this review aims to investigate the impact of oxidative stress, inflammation, mitochondrial dysfunction, and the role of phenolic compounds in managing MAFLD.

## 2. Discussion

### 2.1. Metabolic-Associated Fatty Liver Disease: General Aspects

As pointed out above, the modification of the term NAFLD to MAFLD was proposed due to the augmented knowledge regarding the pathological disease scenario and new therapeutic approaches for individuals, not only in non-alcoholic contexts but all patients presenting fatty liver dysfunction, correlating this dysfunction with other conditions related to metabolic deregulation [57]. Moreover, the definition of MAFLD can recognize hepatic fibrosis better than NAFLD [58,59,60].

MAFLD is profoundly linked to lipid metabolism which involves two pathways, starting with the exogenous pathway, in which the body’s first contact with fat is ingested in the diet [61,62]. Through chylomicrons produced by enterocytes, these lipids are transported through the lymphatic ducts until they reach the bloodstream and then continue the endogenous route through the encountering of these lipoproteins with hepatocytes and the deposition of lipoprotein content in these cells. Then, the triglycerides synthesized in hepatocytes are secreted as VLDL (very-low-density lipoprotein). As it reaches extrahepatic tissues, it loses lipid content and is transformed into IDL (intermediate-density lipoprotein) and LDL-c (low-density lipoprotein cholesterol) [63,64,65,66]. It is possible to better understand the relationship between MAFLD and other diseases through understanding various metabolic processes that occur in mitochondria, such as the tricarboxylic acid cycle (TCA), the β-oxidation of fatty acids, urea synthesis, and respiratory chain [67,68]. When using fuels such as glucose and fatty acids to obtain adenosine triphosphate (ATP), any disturbance in one of these mechanisms can cause severe damage to the cell and, consequently, to the tissue. Mitochondrial changes include a reduction in mitochondrial DNA (mtDNA), structural lesion formation, reduced activity of respiratory chain complexes, and damage to β-oxidation [69,70,71]; therefore, the metabolic repercussions of these changes can be devastating for the body [72,73,74,75,76].

### 2.2. Metabolic-Associated Fatty Liver Disease and Lipid Metabolism

De novo lipogenesis (DNL) is an essential component of the lipid cross-talk between the liver and adipose tissues, maintaining metabolic homeostasis. Imbalance between these tissues is a common feature of conditions associated with obesity, metabolic syndrome, and MAFLD, indicating how important it is to understand how this metabolic pathway contributes to cellular function. In addition, targeting this pathway shows clinical promise in MAFLD treatment [77,78,79,80,81].

On the other hand, excess triglycerides in the liver can decrease VLDL secretion through negative feedback [82]. Increased liver fat accumulation also accelerates inflammatory processes contributing to oxidative stress so that the structure of cell membranes, proteins, and mitochondria can be compromised, reducing the production of this VLDL and balanced distribution of lipids throughout the body [83,84,85,86].

The result of the above-mentioned factors, associated with insulin resistance and hyperglycemia, is liver steatosis. It is related to the abnormal accumulation of triglycerides within parenchymal cells, mainly in the liver [87,88,89,90]. Steatosis occurs when there is a dysfunction in the transport of lipids, with a consequent excessive accumulation of fat in hepatocytes [91], a condition that has a direct link with dyslipidemia and type 2 diabetes mellitus (T2DM) [92], as adipose tissue releases pro-inflammatory cytokines that can interfere with insulin signal transduction pathways. It is also possible to establish a relationship between VLDL levels and DNL, proving that MAFLD is closely related to eating habits [93,94,95,96,97]. The natural process of lipogenesis corresponds to the synthesis and storage of lipids. At the same time, DNL generally occurs in response to excess calories in the diet, synthesizing fatty acids and triglycerides from non-lipid sources such as carbohydrates and proteins. When there is an excess of calories, the liver can increase the production of fatty acids, which are then incorporated into VLDL for transport to other tissues [43,82,97,98,99,100,101,102,103].

The deposit of fats in hepatocytes, the main characteristic of MAFLD, poses a risk of developing NASH [104,105], leading to fibrosis and eventual liver cirrhosis. In this case, normal tissue is replaced by scar tissue, interfering with liver functions, which can result in liver failure and an increased risk of cancer [106]. This is a more advanced condition of steatosis, characterized by the presence of inflammation and damage to liver cells, and deserves attention, as in milder cases, this disease does not cause symptoms, and when more advanced, the most common symptoms are ascites, encephalopathy, mental confusion, bleeding, and a drop in the number of platelets [107,108]. Low levels of albumin, increased amounts of bilirubin, and changes in clotting factors may also indicate liver problems, which can help diagnose steatosis [109,110,111,112,113,114,115,116,117].

In addition to the possible progression to fibrosis and its relation to metabolic syndrome, dyslipidemia, coronary artery disease, inflammation, and oxidative stress, MAFLD may be related to mitochondrial dysfunction, as the pro-inflammatory state caused by an augmented provision of lipids to the liver causes fatty infiltration in hepatocytes, which induces lipid peroxidation and mitochondrial dysfunction [12,15,118,119,120]. 

Polyphenols such as anthocyanin, baicalin, catechin, chlorogenic acid, cichoric acid, curcumin, didymin, ellagic acid, epigallocatechin-3-gallate, gallic acid, hydroxytirosol, kaempferol, luteolin, mangiferin, puerarin, punigalin, quercetin, resveratrol, salvianolic acid, rosmarinic acid, and silymarin can target a variety of pathways related to the physio-pathogenesis of MAFLD pathways and may work as therapeutically significant compounds [41,54,119,121,122,123,124,125].

### 2.3. Metabolic-Associated Fatty Liver Disease, Insulin Resistance, and Oxidative Stress

Cellular respiration is a naturally oxidative process. It occurs through the respiratory chain in mitochondria and is responsible for the transport of electrons and the oxidation of coenzymes in order to produce ATP [126]. The metabolization of fatty acids consequently also occurs through oxidation, in so-called β-oxidation, giving rise to Acetyl-CoA, a molecule responsible for adding acetyl groups in biochemical reactions to metabolize carbohydrates, lipids, and proteins in the production of ATP. When the oxidative process occurs, reactive oxygen species (ROS) are produced [126]. The body has its own mechanism to regulate the amount of ROS produced through compensation by antioxidant enzymes (such as catalase, superoxide dismutase, and glutathione peroxidase) [127,128,129,130,131], in addition to the use of antioxidants such as polyphenols. The mitochondria have an antioxidant system, including enzymes that neutralize part of the ROS produced in respiration [132,133,134,135]. However, when free radicals accumulate, oxidative stress can occur. Fat accumulation is a cause of oxidative stress, especially in visceral tissues [136,137,138,139,140,141]. 

In MAFLD, chronic inflammation and oxidative stress synergize the occurrence of insulin resistance [142,143]. Liver inflammation caused by NASH, associated with the presence of pro-inflammatory mediators such as leptin, resistin, IL-6, and tumor necrosis factor-alpha (TNF-α) and intestinal lipopolysaccharides (bacterial endotoxins), creates an obstacle to insulin signaling pathways, impairing the insulin uptake of glucose in peripheral tissues and the inhibition of liver glucose production. Furthermore, oxidative stress resulting from the accumulation of free fatty acids and lipids in the liver contributes to mitochondrial dysfunction and the activation of inflammatory signaling pathways, increasing insulin resistance [144,145,146]. The elevated production of ROS leads to the oxidation of nucleic acids, proteins, and lipids, compromising cellular function and inducing the production of pro-inflammatory cytokines (TNF-α, IL-6, IL-1β, and TGF-β) [3,147,148]. This inflammatory and stressful environment interferes with the insulin signaling cascade, resulting in an attenuated response of target tissues to insulin and, consequently, the maintenance of hyperglycemia and hyperinsulinemia [149,150].

Insulin resistance can increase the production of advanced glycation ends (AGEs), which aggravate oxidative stress and pro-inflammatory pathways [151,152,153,154]. These pro-oxidant mechanisms also end up contributing to cardiovascular diseases as LDL-c undergoes oxidation, becoming more prone to forming atheroma plaques [155,156]. The heart muscle can also suffer from the dysfunctions mentioned above, which lead to failure and heart tissue damage [154], as inflammatory responses have pathogenic importance by stimulating the production and liberation of inflammatory biomarkers such as IL-6, monocyte chemoattractant protein-1 (MCP-1), and matrix-9 metallopeptidase (MMP-9) [56,157,158,159]. 

### 2.4. Metabolic-Associated Fatty Liver Disease and Inflammation

The relationship between MAFLD and inflammation is of utmost importance in understanding this multifaceted liver condition. Chronic inflammation is essential in the progression of MAFLD, a crucial point of therapeutic intervention. The activation of inflammatory pathways, together with the imbalance of pro- and anti-inflammatory adipokines, contributes to the pathogenesis and transition from simple hepatic steatosis to more severe forms of the disease. Therefore, understanding these inflammatory mechanisms is essential for developing targeted therapeutic strategies, thus mitigating the intensity and preventing the progression of MAFLD [160,161,162,163].

Oxidative stress, as previously mentioned, is closely linked to MAFLD since it triggers pro-inflammatory pathways that can lead to liver diseases or are caused by the evolution of this condition. ROS production can trigger inflammation by activating pro-inflammatory signaling pathways as a homeostatic response to damage and modifications caused to cellular structures in an attempt to repair what has been injured [164,165,166]. In this way, transcription factors are activated, such as nuclear factor kappa B (NF-kB) and mitogen-activated protein kinase (MAPK), responsible for regulating the expression of inflammatory genes [167]. At the same time, hepatocytes and non-parenchymal cells of the liver express Toll-like receptors (TLRs), which recognize molecular patterns associated with lipids and fatty acids. The activation of these receptors start the release of pro-inflammatory cytokines and chemokines, attracting immune system cells to the hepatic region [167,168,169,170,171,172,173].

Oxidative damage also induces the release of cytokines such as pro-inflammatory interleukins, chemokines, and prostaglandins, along with the activation and migration of immune cells to sites that have suffered damage [174]. TNF-α is responsible for the induction of the synthesis of more cytokines, in addition to stimulating the expression of adhesion molecules on endothelial cells. Therefore, the migration of immune system cells to the site of inflammation is favored. IL-6 stimulates the immune response, promoting the activation and differentiation of cells such as T and B lymphocytes and Natural Killer (NK) cells, helping both the innate and acquired immune responses, which, depending on the progression of the condition, can trigger chronic inflammation in the liver tissue, characterized precisely by the presence of macrophages and T lymphocytes, responsible for eliminating inflammatory agents and releasing cytokines [174,175,176,177,178,179].

**Figure 2:**
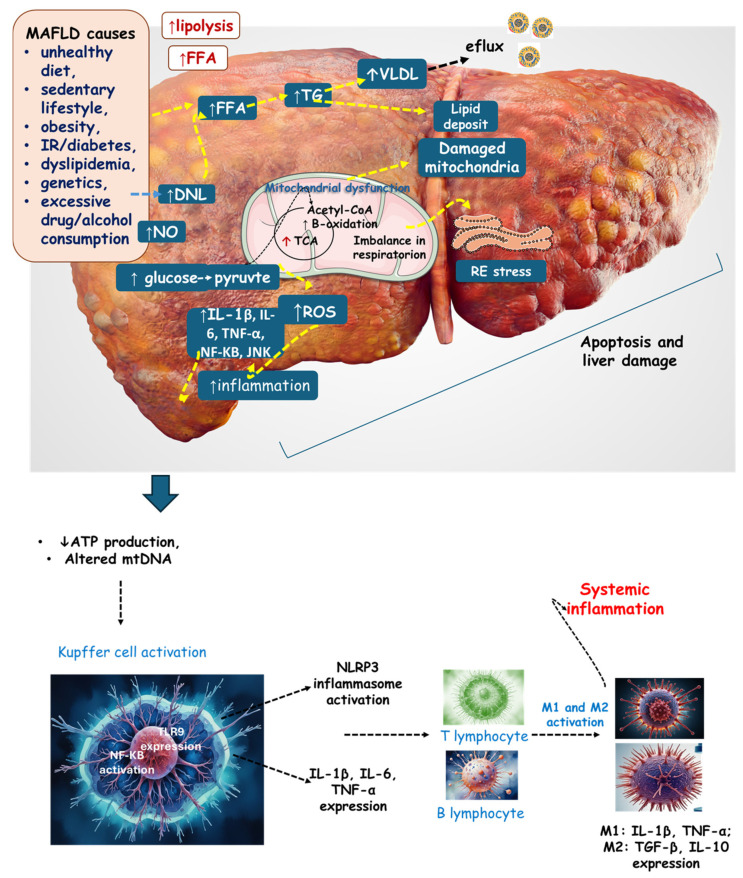
The liver in the context of MAFLD. Lifestyle and metabolic alterations lead to an increased lipolysis of visceral adipose tissue, stimulating de novo lipogenesis, and an increase in FFA and VLDL (and a consequent efflux of this lipoprotein). Increased glucose intake results in increased pyruvate and Acetyl-CoA production, leading to increased TCA activity. Furthermore, there is augmented β-oxidation resulting in mitochondrial dysfunction. The consequences are mitochondrial dysfunction, altered mtDNA, an imbalance in respiration (reduction in ATP production), and RE stress. All these events are related to increased inflammation and ROS, which results in apoptosis and liver damage. Systemic inflammation occurs due to Kupffer cell activation. DNL: de novo lipogenesis; FFA: free fatty acid; IL: interleukin; JNK: c-Jun N-terminal kinase; M2: macrophage; mtDNA: mitochondrial DNA; NF-KB: nuclear factor-KB, NO: nitric acid; NLRP3: NLR family pyrin domain-containing 3; ROS: reactive oxygen species; VLDL: very-low-density lipoprotein; TG: triglyceride; TNF-α: tumor necrosis factor-α; TCA: tricarboxylic acid cycle.

Inflammation becomes chronic due to the persistence of the aggressor stimulus, which can be ROS or growth factors such as transforming growth factor beta (TGF-β), which is responsible for fibroblast proliferation and extracellular matrix deposition [176,180,181]. Fibroblasts produce large amounts of collagen and other proteins, leading to the progression of fibrous tissue. The fibrosis resulting from this process reduces the organ’s original functions due to the replacement of the original tissue with fibrous tissue, mostly composed of collagen, which can thus reduce liver function and cause liver failure [182,183,184,185]. Figure 2 shows a scenario of liver ROS production, inflammation, and mitochondrial dysfunction. 

It is also important to note that inflammation markers are critical in assessing and monitoring MAFLD, as they can suggest both the presence and severity of the disease. These include IL-6, as mentioned above, and the production of C-reactive protein (CRP), which occurs through the binding of IL-6 to specific hepatocyte receptors, a process triggering the stimulation of intracellular signaling pathways, so that the transcription of the CRP gene is induced [186]. In addition to connecting to damaged cells and modulating the immune response due to the activation of the complement system, CRP is an important marker for inflammatory activity [187,188,189,190].

Ferritin also can be considered a marker of inflammation. This protein is related to storing and releasing iron. It can interfere with oxygen transport, energy production, and DNA synthesis. In patients with MAFLD, serum ferritin may be increased because hepatocytes and hepatic macrophages, known as Kupffer cells, in this pro-inflammatory scenario, increase the production of proteins, including ferritin, as an attempt to prevent tissue injury [191,192]. At the same time, this protein helps protect cells from oxidative damage, so its quantity is increased in the abnormal presence of ROS [193,194,195,196]. 

TNF-α is also important in this topic, and high levels are associated with chronic inflammatory diseases. It can trigger insulin resistance by interfering with the correct signaling of this hormone. As already discussed above, this condition is closely related to MAFLD, as the liver’s glucose production through gluconeogenesis increases, contributing to hyperglycemia [197,198,199]. This insulin resistance increases lipolysis in adipocytes, causing an increase in fatty acids in the bloodstream. At the same time, the availability of fatty acids is elevated in the liver, leading to greater hepatic lipogenesis and the accumulation of more lipids. Furthermore, TNF-α can stimulate hepatic stellate cells, responsible for excessive extracellular matrix production and TGF-β activation, leading to liver fibrosis [200,201,202,203].

For all these reasons, treating MAFLD with therapeutic interventions should include changes in diet, the intake of antioxidants and phytochemicals, physical exercise, and the use of medications, which would help decrease inflammatory activity and improve the bad clinical scenario [204,205,206,207,208,209,210,211,212,213].

### 2.5. Metabolic-Associated Fatty Liver Disease and Mitochondrial Dysfunction 

Mitochondria are essential organelles for eukaryotic cells, performing the function of energy production and various metabolic processes. Proper mitochondrial functioning is necessary for cellular homeostasis, which is critical in cellular respiration and ATP production [214,215,216]. Therefore, their structure or function can be related to several consequences (metabolic dysfunction, oxidative stress, and cell death). Mitochondria can have a critical role in the progression or regression of MAFLD [217,218,219]. 

Some authors have shown that in patients with MAFLD, mitochondria had an abnormally activated mitochondrial permeability transition pore, keeping the organelle membranes open for longer due to the intracellular accumulation of free fatty acids [220]. The increase in membrane permeability causes the loss of Ca^2+^ ions, reducing the number of protons that participate in the electron transport chain, resulting in an insufficient production of ATP and an increase in the cytoplasmic concentration of Ca^2+^. This change in gradient concentration inside and outside the organelle can cause changes in the structure of mitochondria and even their destruction, in addition to causing the loss of cytochrome C and coenzyme Q, participants in the respiratory chain [221,222,223,224,225,226].

The loss of membrane potential caused by free radicals also causes a greater quantity of fatty acids to enter the mitochondria, decreasing the activity of proteins in oxidative phosphorylation [227,228] and β-oxidation, resulting in lipotoxic accumulation associated with MAFLD. One of the mechanisms of the self-regulation of metabolic activity is the formation of new mitochondria through the fission and fusion of these organelles [229,230,231]. The first is the process by which mitochondria divide into two units (enabling the exchange of genetic material and the restoration of damaged mitochondria), allowing for the reproduction and renewal of these organelles, an essential process for the production of an adequate supply of mitochondria and sufficient production of energy for the body’s metabolism, in addition to serving as a mechanism for regulating the size and shape of mitochondria, to maintain homeostasis [232,233]. It is possible to observe that in hepatic steatosis, there is an increase in fission and a decrease in fusion, which could be beneficial in normal health conditions [234,235]. However, fission, affected by oxidative stress, accelerates the fragmentation of mitochondrial DNA and stimulates the production of ROS, further contributing to the evolution of MAFLD. Moreover, it has been observed that the new mitochondria formed under these conditions are defective [236,237]. Therefore, it is possible to diagnose and monitor hepatic steatosis through markers of mitochondrial enzymatic activities, ATP production levels, and gene expression related to the fission of new organelles and lipid metabolization capacity so that it is possible from the onset of the disease, carrying out therapeutic interventions in order to reduce the progression of the condition [220,238,239,240,241,242,243]. 

Another element in the mitochondrial dysfunction mechanism is nutrition overload, which accelerates fatty acid oxidation through the TCA and causes ROS overproduction. Excess ROS damages the mitochondrial electron transfer chain (ETC), promoting mitochondrial dysfunction and further cellular apoptosis, inflammation, and liver fibrosis. Beyond that, increased inflammatory mediators, such as NF-κB, IL-6, and TNF-α (related to ROS and inflammation excess), increase the risk of atherosclerosis injuries and damage in the liver vessels [76,244,245,246].

The mitophagy pathway, which is beneficial for removing problematic mitochondria and oxidative toxic byproducts (mt-ROS), is inhibited in MAFLD. In this case, mt-ROS probably increases its concentration inside the cell, promoting a higher level of release of cytochrome C due to ETC activity, causing apoptosis and worsening oxidative stress [247,248,249,250,251].

Excessive free radical production in mitochondria can also trigger a condition known as mitochondrial permeability transition (MPT). In this process, several proteins from the inner mitochondrial membrane, such as the phosphate carrier and the adenine nucleotide translocator (ANT), along with the matrix chaperone cyclophilin D, form a supramolecular structure that acts as a non-specific pore [252,253]. These MPT pores are responsible for dissipating the mitochondrial membrane potential and losing ATP synthesis capacity. It is not only MAFLD that can cause mitochondrial defects; mitochondrial defects can also contribute to MAFLD. For instance, defects or polymorphisms in mitochondrial DNA, like mutations in the gene encoding mitochondrial isobutyryl-coA dehydrogenase or mitochondrial DNA depletion syndromes, can result in excessive lipid accumulation in hepatocytes and the loss of the sirtuin 3 mediator, which can lead to reduced resistance to oxidative stress, and these are some of the various mechanisms that can contribute to the pathology [254,255,256].

In MAFLD, besides the dysfunction in mitochondrial metabolism during fat accumulation, the endoplasmic reticulum (ER) also plays a role in metabolite exchange through complex polymeric protein structures such as mitochondrial-associated membrane proteins (MAM) [215,257,258,259,260]. When there is an imbalance in ER homeostasis or energy deficiency, the ER is activated by the unfolded protein response (UPR), leading to a reduction in glutathione (GSH). This imbalance in the distribution between GSH and oxidized glutathione (GSSH) induces mitochondrial stress and results in an impaired regulation of mt-ROS production, leading to an increase in its concentration, which is a crucial factor in the enhancement in oxidative stress [261,262].

**Figure 3:**
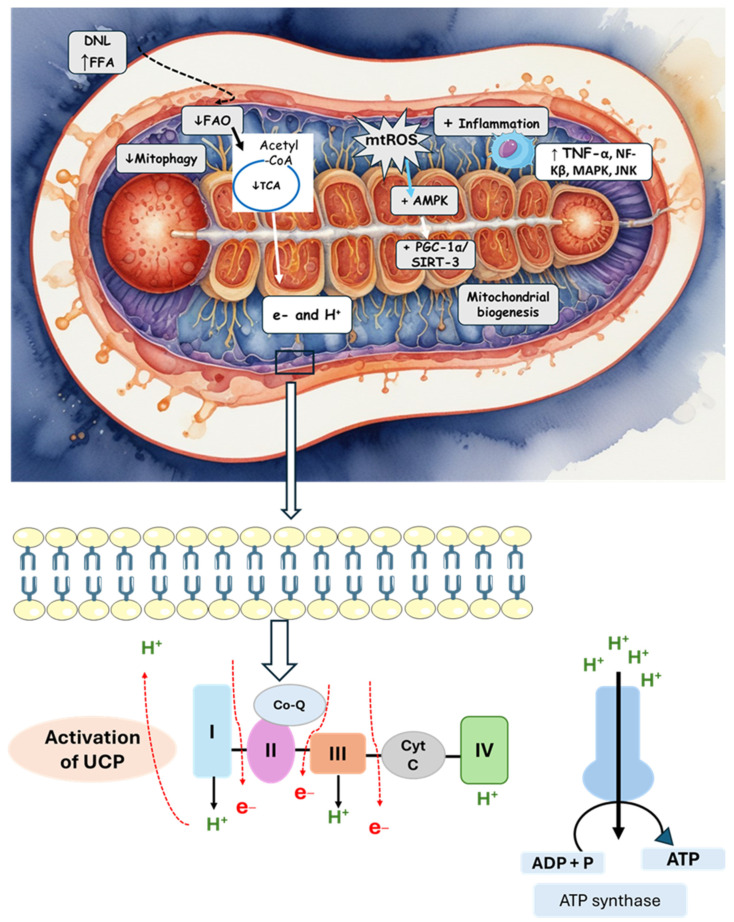
The activation of DNL and an increase in FFAs lead to mitochondrial alterations and an increase in oxidative stress and inflammation. The stimulation of the mitochondrial membrane permeability transition pore is also observed by mitochondrial alterations and the deposit of fatty acids. There is stimulation in the activity of inner membrane proteins, leading to a reduction in ATP production. Mitochondrial gene mutation (mt-DNA) also activates uncoupling proteins. AMPK: AMP-activated protein kinase; CoQ: coenzyme Q; Cyt C: cytochrome C; DNL: de novo lipogenesis; FAO: fatty acid oxidation; FFA: free fatty acid; PGC1α: peroxisome proliferator-activated receptor-γ coactivator 1-α; JNK: c-Jun N-terminal kinase; NF-KB: nuclear factor kappa B; SIRT3: sirtuin 3; TCA: tricarboxylic acid cycle; TNF-α: tumor necrosis factor-α; UCP: uncoupling protein.

Figure 3 summarizes the mechanisms of mitochondrial dysfunction.

### 2.6. Polyphenols and Metabolic-Associated Fatty Liver Disease

**Figure 4:**
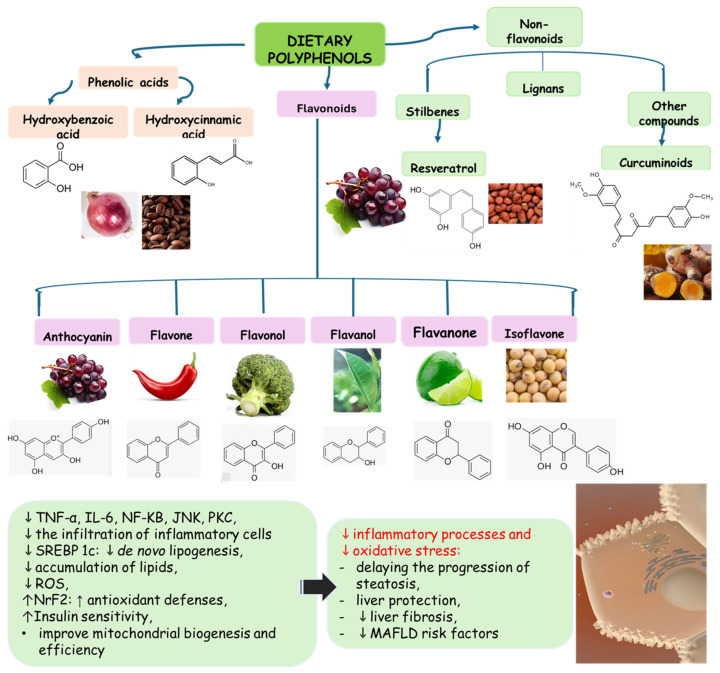
Polyphenols: classification and origin. Polyphenols are found in many fruits and vegetables and can be separated into phenolic acids, flavonoids, and non-flavonoids. Phenolic acids can be found in onion, tea, and coffee; flavonoids in grapes, pepper, broccoli, green tea, lemon, and soy; and non-flavonoids in grapes, peanut skin, and *Curcuma longa*. These compounds can protect the liver since they can reduce the risks for MAFLD, such as oxidative stress, inflammation, and lipid deposits. IL: interleukin; JNK: c-Jun N-terminal kinase; MAFLD: Metabolic-Associated Fatty Liver Disease; NF-KB: nuclear factor kappa B; Nrf2: nuclear factor erythroid 2-related factor 2, PKC: protein kinase C; ROS: reactive oxygen species; SREBP-1c: Sterol regulatory element-binding protein 1c.

Polyphenols are bioactive compounds of plant origin that, when ingested, act as natural antioxidants [54,55,263,264,265,266,267,268,269,270,271,272,273,274,275]. They are present in foods according to color and are responsible for vegetables’ characteristic aromas and flavors. In plants, phytochemicals have the role of resistance to infections by bacteria, fungi, and viruses, as well as the consumption of insects and other animals. For humans, they are known for their antioxidant and anti-inflammatory actions. Among these plant biocompounds, some phenols and polyphenols can also help prevent several health conditions related to oxidative or inflammatory processes. Dietary polyphenols can include phenolic acids, flavonoids, and non-flavonoids. The most known flavonoids are anthocyanins, flavones, flavanol, and isoflavones. Among non-flavonoids are stilbenes and lignans (Figure 4). These compounds are also known to strengthen immunity, regulate the body’s hormonal activity, and promote mitochondrial health [273,276,277,278,279,280,281,282]. Table 1 shows the main polyphenols related to benefits for liver conditions. 

Polyphenols can protect mitochondria from oxidative stress due to the potential antioxidant effects essential for the proper functioning of these organelles. Furthermore, some interfere with metabolic processes related to energy production by regulating the activity of enzymes involved in the formation of ATP and promoting the process of mitochondrial renewal and biogenesis [283,284,285,286,287,288,289].

Natural or processed products, when added with polyphenols, can increase their antioxidant and anti-inflammatory power, bringing benefits to the consumer in terms of preventing health conditions of an oxidative or pro-inflammatory nature such as cardiovascular diseases, inflammatory diseases, and cancer [290].

However, the bioavailability of these polyphenols may be insufficiently low to reach an effective plasma level to produce the desired effects. Therefore, new pharmaceutical formulations have been developed, such as nanoparticles, nanoemulsions, nanomicelles, and preparations that increase absorption [125,291,292].

**Table 1:** Some polyphenols related to the improvement in MAFLD risk factors.

Bioactive Compound	Molecular Structures	Plant Rich in the Biocompound	Part of the Plant	Effects	References
Anthocyanin	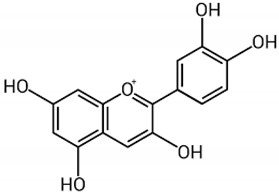	Berries, strawberries, and grapes	Leaves, flowers, fruits, and roots	Antioxidant and anti-inflammatory, lipolysis induction, modulation of lipoprotein metabolism and PPARs	[293,294,295]
Baicalin	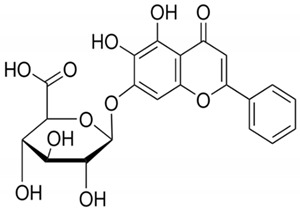	*Scutellaria baicalensis*	Roots	Anti-inflammatory, antioxidant, and hepatoprotective	[296,297,298]
Catechin	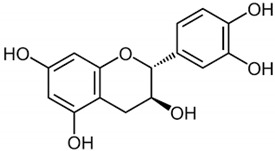	*Camellia sinensis*	Leaves	Anti-inflammatory, antioxidant, and hepatoprotective	[299,300]
Chlorogenic acid	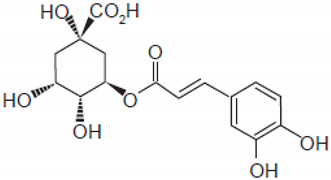	Green coffee	Roots	Anti-inflammatory, antioxidant, and hepatoprotective	[301,302,303]
Cichoric acid	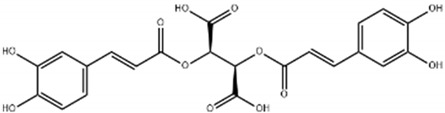	*Cichorium intybus*	Leaves, flowers, and roots	Antilipogenesis, prevention of lipid accumulation and fibrosis	[304,305,306]
Curcumin	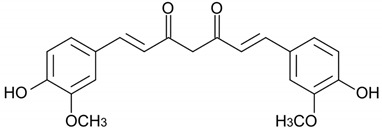	*Curcuma longa*	Rhizome	Anti-inflammatory and antioxidant	[307,308,309,310,311,312,313,314,315,316,317]
Didymin	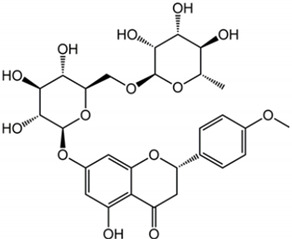	Citrus fruits	Fruit	Anti-inflammatory and antioxidant	[318,319,320]
Epigallocatechin-3 gallate	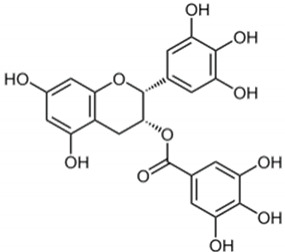	*Camellia sinensis*	Leaves	Antioxidant and anti-inflammatory	[321,322]
Kaempferol	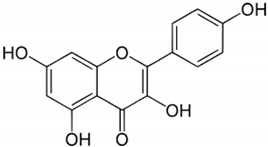		Leaves and stem	Antioxidant, anti-inflammatory, and improves insulin resistance	[323,324]
Luteolin	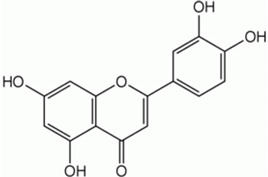	Celery, peppers, carrots	Leaves and seeds	Antioxidant and anti-inflammatory	[90,325]
Mangiferin	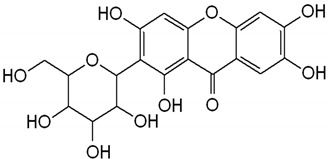	Mango	Leaves, roots, and stem	Regulation of glucose and lipids metabolism	[48,326,327]
Puerarin	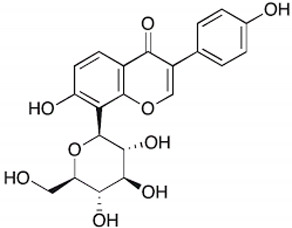	*Pueraria lobata*	Roots	Antioxidant and anti-inflammatory	[328,329]
Punicalagin	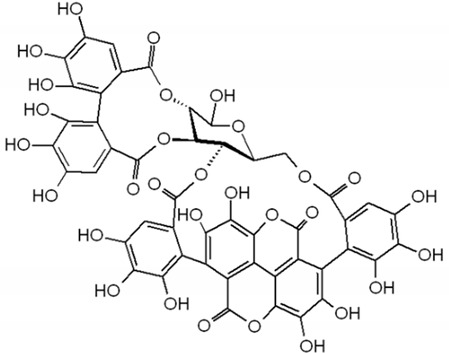	*Punica galum*	Shells and seeds	Decreases lipid accumulation and increases gene expression levels of fatty acid beta-oxidation pathways	[330,331]
Quercetin	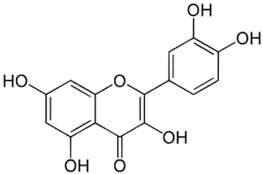	*Evodiae fructus*	Bark, leaves, flowers, seeds, and shoots	Improvement in insulin resistance, modulation of lipid metabolism, reduces inflammation and oxidative stress	[332,333,334]
Resveratrol	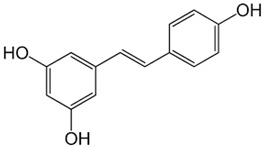	Red grapes and peanuts	Grape and peanut skin	Mitochondrial biogenesis and synthesis; antioxidant and anti-inflammatory	[335,336,337,338]
Rosmarinic acid	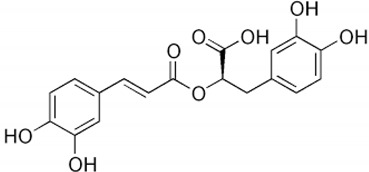	*Salvia rosmarinus*	Leaves	Antioxidant and anti-inflammatory	[339,340]
Silymarin	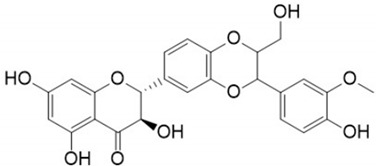	*Silybum marianum*	Leaves, fruit, and seeds	Reduction in liver injury and lipid accumulation; insulin resistance improvement	[341,342,343,344]

#### 2.6.1. Anthocyanins

Anthocyanins are flavonoids responsible for the reddish color of foods such as red fruits and vegetables such as red cabbage, purple potatoes, and eggplant. Studies carried out on cells derived from hepatocellular carcinoma indicate that they can reduce the accumulation of lipids in hepatocytes by inhibiting lipogenesis. At the same time, they promote lipolysis and reduce oxidative stress through the activation of AMPK, being able to act agonistically on peroxisome proliferator-activated receptors (PPARs) in liver cells. PPARs act as transcription factors of lipid metabolism, especially PPAR-α, widely found in the liver, which regulates mitochondrial and peroxisome β-oxidation and plays an important role in lipoprotein synthesis [293,294,295].

#### 2.6.2. Baicalin

By modifying signaling pathways, baicalin (a flavonoid derived from *Scutellaria baicalensis*) can potentially reduce NAH, hepatic steatosis, and MAFLD. It acts on nuclear factor kappa B (NF-κB), thus reducing inflammation in the liver, a crucial factor in the development of liver diseases. This polyphenol can also act on transforming growth factor beta 1 (TGF-β1)/SMAD3, reducing liver fibrosis. Baicalin intensifies sirtuin 1 (SIRT1) by upregulating lipid metabolism. In addition, it inhibits p38/MAPK and has the ability to reduce oxidative stress and programmed hepatocyte death [296,298]. Baicalin regulates MERTK +/hi M2c derived from mononuclear cells (MNCs), demonstrating a role in modulating the liver’s immune response and attenuating chronic hepatic inflammation. The interaction of baicalin with the enzyme carnitine palmitoyltransferase 1 (CPT1) promotes the oxidation of fatty acids, favoring the reduction in lipid accumulation in the liver [297]. 

#### 2.6.3. Catechin

Catechins, largely found in *Curcuma longa* (saffron) and *Camellia sinensis* (green tea) [345], act similarly to anthocyanins through an indirect activation of PPARα [299], as they inhibit oxidative and inflammatory activity, responsible for decreasing the expression of this receptor; they can also elevate the gene expression of proteins involved in lipid metabolism and modulate signaling pathways, such as the AMPK pathway [346]. Their antioxidant activity occurs through the neutralization of free radicals by donating electrons while at the same time having the property of binding to metal ions, preventing Fenton reactions—the decomposition of hydrogen peroxides catalyzed by FeII and the production of HO radicals and the generation of FeIV, highly oxidizing products [347]. Catechin may also increase the activity of antioxidant enzymes by interfering with nuclear factor erythroid 2-related factor 2 (Nrf-2) translocation [300]. 

#### 2.6.4. Chlorogenic Acid

Chlorogenic acid, found in green tea, fruits, and green coffee [22,348,349], is capable of acting on the intestine–liver axis; it has antilipogenic and anti-inflammatory action and helps regulate the intestinal microbiota. It may be related to the degradation of fatty acids through the activation of hepatic autophagy, binding to ALKBH5 (demethylase Alk B homolog 5) and preventing its action of removing methyl groups from position 6 of the adenine of messenger RNA (m6A). This process compromises the gene expression of liver cells, promoting autophagy to reduce hepatic steatosis [303]. It is also related to the improvement in the expression of carnitine palmitoyltransferase (CPT-1), responsible for conjugating long-chain fatty acids to carnitine in the mitochondria so that β-oxidation occurs [301,302].

#### 2.6.5. Cichoric Acid

The chicory plant (*Cichorium intybus*), Astraceae family, is a source of vitamins, phenolic acids, and cichoric acid) [350]. Cichoric acid plays an important role in reducing hepatic steatosis, as it reduces the expression of lipogenic actors, such as SREBP-1c, DGAT1, FAS, and SCD-1, and also of inflammatory factors such as IL-6, IL-1b, NF-κB, and TNF-α. It is known that advanced steatosis can cause fibrosis, and cichoric acid also prevents TGF-β and the development of type I and type III collagen in the liver [351]. In a study where HePG2 cells were treated with palmitate, it was observed that fish oil, together with cichoric acid, significantly reduced lipid accumulation through the AMPK-mediated stimulation of PPAR-α [319]. 

#### 2.6.6. Curcumin

Curcumin is a polyphenol belonging to curcuminoids, which are compounds of the ginger family. It is found in the rhizome of *Curcuma longa*. Curcumin is associated with a reduction in body weight, improves insulin resistance, reduces lipid levels, reduces inflammation and oxidative stress, and can improve liver disease (showing a decrease in hepatic fat levels and a reduction in serum aspartate aminotransferase and alanine aminotransferase levels) [125,311,316,352]. It can inhibit cytotoxins and cyclooxygenase (COX) and lipoxygenase (LOX) enzymes [353,354], responsible for producing prostaglandins and leukotrienes, respectively, which are mediators that contribute to the inflammatory process. At the same time, curcumin also plays a role in reducing the production of free radicals in some ways, such as donating electrons to these radicals to become stable or increasing the activity of the body’s natural antioxidant enzymes, such as superoxide dismutase and glutathione peroxidase. In this way, they can contribute to the treatment of MAFLD along with lifestyle changes [355] and may also prevent the development of liver fibrosis, resulting in a 3- to 5-fold higher chance of resolution in hepatic steatosis [356]. 

It has also been demonstrated that the association of curcumin with resveratrol has led to a synergistic effect by attenuating MAFLD, and this result may be, at least in part, associated with the modulation of the Hypoxia-inducible factor 1 (HIF-1) signaling pathway. HIF can modulate lipid metabolism in a particular way in the liver tissue by sensing the cellular microenvironment under different conditions. In a low-oxygen environment, HIF-1 stimulates the uptake and utilization of fatty acids and can elevate lipogenic gene expression, therefore augmenting lipid accumulation in the liver [357].

#### 2.6.7. Didymin

Didymin, a flavonoid identified in citrus fruits, has antioxidant and anti-inflammatory action, making it suitable for use in MAFLD as a therapeutic intervention. In experiments, it was noted that didymin results in the activation of Sirt1, a sirtuin that regulates energy metabolism and the inflammatory response. Sirt1 activation is linked to the inhibition of the TLR4/NF-κB pathway (an inflammatory pathway that determines the progression of MAFLD), showing that didymin can attenuate hepatic inflammation and oxidative stress [358]. Furthermore, it can suppress the PI3K/Akt pathway, demonstrated by the decrease in the phosphorylation levels of PI3K and Akt, which modulates insulin resistance and lipid accumulation in hepatocytes correlated with MAFLD. Thus, its therapeutic role is a natural intermediary one that adds to existing therapeutic strategies [318]. 

#### 2.6.8. Epigallocatechin-Gallate (EGCG)

EGCG is the major active compound found in green tea and has been linked to a reduction in obesity and an improvement in metabolic parameters. A study aiming to evaluate the effects of this compound on lipolysis, obesity, and the browning of human white adipocytes showed that EGCG can significantly reduce systolic and diastolic blood pressure (*p* < 0.05), fasting plasma triglyceride levels (*p* < 0.05), and serum kisspeptin levels (*p* < 0.05) after eight weeks of supplementation [359].

#### 2.6.9. Kaempferol

Kaempferol, a flavonoid found in foods such as broccoli, kale, green tea, and apples, has therapeutic properties in the fight against numerous liver diseases. This polyphenol promotes antioxidant and anti-inflammatory results, which are essential for liver protection [324,360]. It intensifies the action of superoxide dismutase, an antioxidant enzyme, and catalase [361]. This compound acts on PI3K/AKT signaling, improving insulin receptivity. Kaempferol can prevent H_2_O_2_-induced oxidative stress in the production of nitric oxide (NO) coordinated by HepG2 and lipopolysaccharides (LPSs) in RAW264.7 cells. It also acts by reducing the production of free oxygen radicals, rebuilding the redox balance, and preventing the production of exaggerated NO, a mediator of inflammation caused by exposure to LPSs [323]. Kaempferol intensifies the action of the activated protein kinase AMPK, favoring beta-oxidation, which reduces the formation of lipids in the hepatic region [362]. These consequences are important to prevent the progression of MAFLD to NASH. The compound has antiapoptotic and anti-necroptotic capabilities, protecting hepatocytes from predisposed death. Additionally, kaempferol is related to the restriction of cyclooxygenase and lipoxygenase enzymes [363]. 

#### 2.6.10. Luteolin

Luteolin, a flavonoid identified in vegetables (celery, peppers, carrots, and some medicinal herbs), has been highlighted for its therapeutic role in liver diseases. It exhibits anti-inflammatory and antioxidant results, which are essential in protecting the liver against oxidative and inflammatory damage related to fat accumulation [364]. Studies show that luteolin can significantly reduce the infiltration of inflammatory cells in liver tissue, in addition to attenuating the amount of liver enzymes and lipids in the liver, conditions that influence the progression of MAFLD. Luteolin prevents oxidative damage by neutralizing ROS and enhancing the functioning of endogenous antioxidant enzymes, which detoxify free radicals [365]. Furthermore, luteolin improves insulin sensitivity by regulating the PI3K/AKT/FoxO1 signaling pathway, which is necessary for glucose capture by hepatocytes and muscle cells [366]. The intensification of this pathway exacerbates the translocation of cell membrane proteins, helping the intake of glucose and thus reducing blood glucose. Simultaneously, luteolin intensifies the oxidation of fatty acids by activating AMP-activated protein kinase (AMPK), an enzyme that allows for the beta-oxidation of fatty acids in mitochondria, improving mitochondrial functioning and decreasing hepatic lipogenesis [367].

#### 2.6.11. Mangiferin

Mangiferin, found especially in mangoes and other plants, has antioxidant properties, which is why it helps improve the condition of MAFLD [368]. This polyphenol can reverse the translocation of GLUT4 in the membrane, consequently interfering in the modulation of liver glucose and lipid metabolism, especially in MAFLD [326]. Mangiferin influences the AMPK protein, causing the activation of AKT phosphorylation. This activation is related to the regulation of pantothenate and CoA biosynthesis, which is essential for hepatic lipid metabolism [368] and can also modulate the NLRP3 inflammasome (a protein complex involved in chronic liver inflammation in MAFLD), suppressing its activation. These findings show that mangiferin acts on dysfunctional metabolic aspects characteristic of NASH and in the control of hepatic inflammation [48]. 

#### 2.6.12. Puerarin

Puerarin, a bioactive compound found in *Pueraria lobata* roots, aroused interest as a potential therapy for MAFLD [329]. Researchers suggest that puerarin has antioxidant and anti-inflammatory properties that may help reduce fat accumulation in the liver and mitigate liver inflammation, two crucial components of MAFLD [328,369]. Furthermore, preclinical studies have indicated that puerarin can regulate lipid and glucose metabolism, helping to improve insulin sensitivity and reduce triglyceride and cholesterol levels, factors that are often dysregulated in patients with MAFLD [370]. 

In a *Salmonella enterica*-infected chick model, puerarin protected against infection and improved liver morphology, inflammatory indices, and antioxidant capacity in chicks. Moreover, it significantly decreased the levels of hepatocellular carcinoma markers in the liver [371]. In rats, puerarin reduced liver fibrosis through the signaling pathway mediated by TGF-β/extracellular signal-regulated kinase ½ (ERK1/2), inhibiting hepatic stellate cell stimulation and excessive collagen deposition in liver fibrosis [372].

#### 2.6.13. Punicalagin

Punicalagin is a flavonoid found mainly in *Punica galum* [373] and positively affects the functioning of mitochondria. A study carried out on maturing adipocytes showed that the presence of punicalagin decreased lipid accumulation and significantly increased the gene expression levels of fatty acid beta-oxidation pathways such as peroxisome proliferator-activated receptor γ (PPARγ)C1α, uncoupling protein-1 (UCP-1), and PR domain-containing 16 (PRDM-16), increasing mitochondrial efficiency [330]. Thus, the increase in lipolysis and the decrease in hypertrophic adipocytes reduce the secretion of adipokines, associated with obesity and the inflammation of vascular cells [143]. 

#### 2.6.14. Quercetin

Quercetin, found in the rhizome of *Evodiae fructus*, is a phytochemical that helps treat MAFLD and reduce cancer [374]. This flavonoid acts in terms of AMPK, improving insulin resistance and helping with lipid metabolism to reduce liver fat [375]. Quercetin may also reduce inflammation caused by MAFLD by inhibiting the release of inflammatory biomarkers such as TNF-α and IL-6 [375], along with antioxidant actions, neutralizing free radicals and reducing oxidative stress [376]. 

There is a combination of different clinical and biochemical factors that lead to metabolic dysregulation. Quercetin intake can significantly decrease fasting blood glucose and systolic blood pressure [377]. 

#### 2.6.15. Resveratrol

A well-known example is resveratrol, which comes from grapes, red fruits, peanuts, and wine. It exhibits antioxidant properties associated with the production of mitochondria. Resveratrol is a non-flavonoid polyphenol [378] capable of improving mitochondrial biogenesis, acting on the main effectors of biogenesis, such as the peroxisome proliferator-activated coactivator γ-1α (PGC-1α), sirtuin 1 (SIRT1), adenosine monophosphate protein kinase (AMPK), α-related receptor estrogen (ERR-α), telomerase reverse transcriptase (TERT), mitochondrial transcription factor A (TFAM), and nuclear respiration factors 1 and 2 (NRF-1, NRF-2) [379,380,381]. A study carried out in mice also revealed that resveratrol supplementation significantly increased the activity of SIRT1 and PGC-1, improving the efficiency of mitochondrial synthesis [335]. 

#### 2.6.16. Rosmarinic Acid

Rosmarinic acid, found in *Salvia rosmarinus* (rosemary) and *Prunella vulgaris* [57,382], has antioxidant effects through the modulation of signaling pathways. This polyphenol acts on MAPKs, reducing oxidative stress and hepatic inflammation. It acts on the activation of quinone acceptor oxidoreductase 1 (NQO1) and Nrf2. The increase in MAPKs and Nrf2 reduces the effects related to liver disease. Rosmarinic acid acts on the negative regulation of YAP1 and TAZ, related to the activation of PPARγ and PGC-1α, regulating lipid metabolism and providing hepatic homeostasis [339,340,383]. 

#### 2.6.17. Silymarin

Silymarin, a group of flavonolignans extracted from milk thistle (*Silybum marianum*), has motivated scientific interest due to its therapeutic capacity in several liver diseases, including MAFLD and NASH). Silymarin has beneficial effects through its antioxidant and anti-inflammatory characteristics. Studies indicate that silymarin improves liver function by reducing oxidative stress and inflammation through the formation of glutathione peroxidase, which reduces glutathione to hydrogen peroxide and water, providing the recovery of damaged liver cells [384].

**Figure 5:**
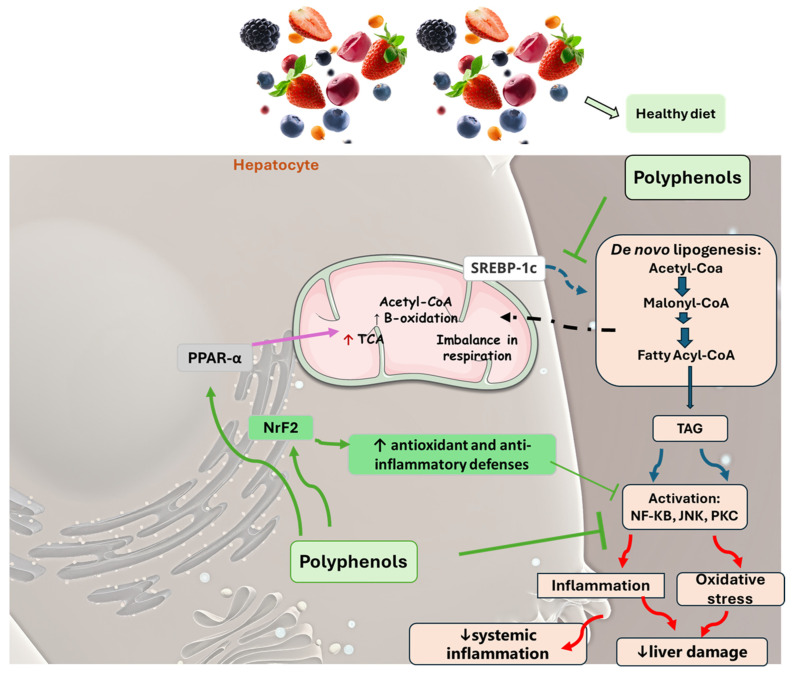
The main mechanisms of action promoted by phenols in MAFLD. A salubrious diet with an increased consumption of fruits and vegetables elevates the intake of polyphenols. These phytochemicals can inhibit liver cellular damage associated with MAFLD through varied mechanisms that may include a decrease in de novo lipogenesis due to the downregulation of SREBP-1c, elevating β-fatty acid oxidation through PPAR α upregulation, ameliorating insulin sensitivity, and reducing oxidative stress and inflammation processes. This scenario is related to a reduction in liver damage and systemic inflammation. JNK: c-Jun N-terminal kinase; NF-KB: nuclear factor kappa B; Nrf2: nuclear factor erythroid 2-related factor 2, PKC: protein kinase C; PPAR-α: peroxisome proliferator-activated receptor gamma; SREBP-1c: Sterol regulatory element-binding protein 1c; TCA: tricarboxylic acid cycle; TAG: triglyceride.

In some animal models and clinical investigations, it was observed that silymarin can reduce the accumulation of lipids in the liver, improve insulin sensitivity, and modulate metabolic pathways related to lipid and glucose metabolism [385]. These results are important for treating MAFLD, in which insulin resistance and metabolic dysfunction play central roles. By improving liver integrity and function, silymarin, in addition to delaying the progression of steatosis to more severe forms, such as NASH, can also reverse initial liver disorders, proving to be a promising treatment for controlling these chronic liver diseases [103]. Figure 4 shows the main polyphenols that can be obtained from the diet, and Figure 5 shows the primary mechanism of action of these compounds in the liver.

### 2.7. Effects of Polyphenols in MAFLD: Results of Clinical Trials

Table 2 shows some clinical trials that investigated the effects of polyphenols in MAFLD. Although some results are controversial, in general, they showed that these compounds can be effective in reducing or preventing risk factors for liver conditions.

Polyphenols are normally related to antioxidant and anti-inflammatory effects; thus, they can be related to different actions in several metabolic and physiological pathways, leading to characteristics that can improve liver tissue damage and function which are observed in MAFLD and its complications.

Besides the suggestion that other clinical trials with a more homogeneous population and a higher number of participants should be performed with the phytocompounds considered in this review, we can say that these phytocompounds can act as preventive compounds or can provide natural treatment and complement existing treatments for MAFLD and other liver conditions such as steatosis. In summary, the results of the included clinical trials show that these compounds, in comparison to placebo, can achieve the following:

- Significantly reduce gamma-GT, AST, and ALT;
- Reduce body weight, BMI, waist circumference, adipose visceral indices, and visceral and subcutaneous abdominal fat mass;
- Decrease plasma total cholesterol, triglycerides, and LDL-c;
- Reduce the plasma concentrations of atherogenic oxidized LDL-c;
- Reduce glycated hemoglobin, glycemia, insulin resistance, and the HOMA index;
- Reduce plasma leptin levels (as well as the leptin–adiponectin ratio);
- Reduce urinary 8-isoprostane excretion;
- Reduce the induction of NFKB;
- Reduce serum cytokeratin-18 and kisspeptin levels;
- Reduce the levels of pro-inflammatory interleukins such as IL-1β, IL-6, IL-18, and TNF-α;
- Decrease the mRNA expression of NLRP3 inflammasome (caspase-1, IL-1β, and IL-18) in peripheral blood mononuclear cells;
- Reduce systolic and diastolic blood pressure;
- Improve HDL-c and adiponectin levels;
- Improve the liver-to-spleen computed tomography attenuation ratio;
- Improve flow-mediated dilation and carotid intima–media thickness;
- Decrease liver fat content, the steatosis index, and the level of fibrosis;
- Improve fibrogenesis markers.

## 3. Conclusions and Future Directions

For MAFLD, the cornerstone of current treatment strategies involves significant lifestyle modifications. These typically include dietary changes and increased physical activity. While these approaches can be effective, the incorporation of polyphenols into these strategies offers exciting potential for enhancing therapeutic outcomes. Polyphenols, known for their antioxidant and anti-inflammatory properties, can complement traditional interventions and provide additional benefits in managing MAFLD (In patients with hypercholesteremia, 70% of them do not respond adequately to statins. For these reasons, using polyphenols in these conditions may bring to light a new direction [412,413]). However, to fully realize the potential of polyphenols in this context, future research needs to explore their impact on various metabolic pathways and liver function biomarkers more comprehensively. Understanding how polyphenols affect these processes could reveal mechanisms through which they influence liver health, potentially leading to novel therapeutic strategies. Moreover, while the Phytochemical Index serves as a valuable tool for assessing dietary polyphenol content, there is an opportunity to refine and enhance its application in clinical settings. By developing more precise and clinically relevant measures of polyphenol intake and their biological effects, we can better guide dietary interventions and tailor recommendations for individuals with MAFLD. 

Firstly, focusing on the bioavailability and metabolic conversion of polyphenols is essential. Understanding how different polyphenols are absorbed, metabolized, and converted into their active forms will provide a more accurate reflection of their potential benefits for liver health. Since polyphenols vary significantly in these aspects, this refinement will ensure that the index accounts for not just the quantity of polyphenols consumed but also their efficacy within the body. Expanding the index to include a broader range of polyphenol compounds is also crucial. By developing a detailed profile that encompasses a variety of polyphenols known to impact liver health, the index can offer a more comprehensive measure of dietary intake. Employing advanced analytical techniques like liquid chromatography–mass spectrometry (LC-MS) will enhance the accuracy of these measurements and help identify which specific polyphenols are the most beneficial for MAFLD management.

Additionally, personalizing the Phytochemical Index based on individual genetic and microbiome profiles is another important step. Variations in genetic makeup and gut microbiota can significantly influence polyphenol metabolism and efficacy. By integrating these personalized data into the index, dietary recommendations can be tailored to individual needs, optimizing the benefits of polyphenols for managing MAFLD. 

To ensure that the refined index is practical and reliable, it should also be validated through rigorous clinical trials. These studies would assess how polyphenol intake, as guided by the index, impacts liver biomarkers and clinical outcomes in MAFLD patients. Clinical validation will provide the necessary evidence to support the index’s effectiveness and its integration into standard clinical practice. Furthermore, developing practical measurement tools will enhance the application of the Phytochemical Index in everyday settings. For example, mobile health apps or digital platforms could be designed to track polyphenol intake and provide real-time feedback. Such tools would help patients adhere to dietary recommendations and make informed choices about their diet, facilitating a better management of MAFLD. 

Several critical research areas also warrant exploration to fully harness the potential of polyphenols. As an example, research into polyphenols and their effects on immune system function could provide significant benefits for managing MAFLD. Understanding how polyphenols influence immune cell markers in subjects with MAFLD could lead to strategies for reducing liver inflammation in these patients, potentially slowing or reversing liver damage. Studying how polyphenols affect immune cell activation in MAFLD could reveal methods to prevent or reduce liver damage caused by immune responses. Genetic research could also identify polymorphisms that affect individual responses to polyphenols, potentially through genome-wide association studies (GWASs). This research would facilitate personalized nutrition approaches by tailoring polyphenol interventions based on genetic predispositions, optimizing therapeutic outcomes.

In this scenario, RNA-based assays, such as transcriptomic studies using RNA sequencing, could illuminate the molecular mechanisms by which polyphenols alter gene expression in the MAFLD liver. This research could uncover specific genes and pathways influenced by polyphenols, providing a clearer understanding of their role in cholesterol metabolism and liver health. Advancements in nanotechnology also hold promise for enhancing polyphenol delivery and effectiveness. Developing nanocarriers for controlled release and targeted action could improve the bioavailability of polyphenols, maximize their therapeutic benefits, and minimize potential side effects. 

Moreover, exploring the synergistic effects of polyphenols in combination with established medications could also lead to novel treatment strategies against MAFLD. Research could focus on how polyphenols interact with statins, other lipid-lowering agents, or diabetes medications in the context of MAFLD to enhance efficacy or reduce adverse effects, offering new insights into optimizing combination therapies.

However, since all novel interventions start with preclinical research, clinical trials are essential to translate these findings into practical clinical applications. These trials should include diverse populations and consider long-term outcomes to assess efficacy, safety, and optimal dosages. Evaluating different forms of polyphenol intake—such as supplements, functional foods, or fortified diets—will also be crucial. Integrating metabolomic and proteomic analyses into research could provide a comprehensive understanding of how polyphenols influence metabolic pathways and protein expression in the realm of clinical research.

Research should also consider how environmental factors and lifestyle choices interact with polyphenol consumption in MAFLD conditions. Cohort studies exploring the effects of diet, microbiome composition, and exposure to environmental toxins on polyphenol efficacy could provide additional insights into optimizing their use against MAFLD.

In summary, while current evidence supports the beneficial role of polyphenols in managing cholesterol and metabolic diseases, advancing our understanding through targeted research is essential. By employing advanced technologies, conducting rigorous clinical trials, and exploring synergistic effects, we can unlock the full potential of polyphenols. This comprehensive approach promises to enhance patient outcomes and contribute significantly to advancements in public health.

## Figures and Tables

**Table 2:** Clinical trials showing the effects of some polyphenols in liver conditions (MAFLD).

Reference	Model/Country	Population	Intervention/Comparison	Outcomes	Side Effects
**Anthocyanin**					
[386]	Randomized, double-blind, placebo-controlled pilot trial	33 patients (20 in the anthocyanin group, 13 in the control group	320 mg/day or placebo for 12 and 24 weeks	There was a higher reduction in ALT in the anthocyanin group than in the placebo group (−19.1% vs. −3.1%, *p* = 0.02).	NR
[387]	Case–control and a randomized controlled intervention trial	312 MAFLD patients	320 mg/day or placebo for 12 weeks	The mRNA expression of NLRP3 inflammasome components (caspase-1, IL-1β, and IL-18) in PBMCs and also the plasma levels of IL-1β and IL-18 were dramatically decreased in treated NAFLD patients compared with controls.	NR
**Catechin**					
[388]	Randomized, double-blind study	17 patients with MAFLD	Participants consumed green tea with high-density catechins, low-density catechins, or a placebo for 12 weeks.	All participants in the high-density catechin group had a significantly improved liver-to-spleen computed tomography (CT) attenuation ratio compared to the other groups; they also had reduced body fat, AST and ALT, and urinary 8-isoprostane excretion. In conclusion, the use of 700 mL/d green tea with >1 g catechin improved liver fat content and inflammation by decreasing oxidative stress.	NR
**Chlorogenic acid and luteolin**					
[389]	Randomized, double-blind, placebo-controlled. Italy, Spain, Poland, USA.	100 individuals with MetS. (28♂, 22♀, 63 ± 11 y); 50 randomized (26♂, 24♀, 63 ± 8 y)	50 subjects were randomized to Altilix^®^ (supplement with chlorogenic acid and luteolin)/6 months	There was a significant amelioration in the treated group compared to placebo in most parameters evaluated, including body weight, waist circumference, glycated hemoglobin, lipid plasma levels, liver transaminases, flow-mediated dilation, and carotid intima–media thickness. Supplementation with Altilix^®^ improved hepatic and cardiometabolic parameters in individuals with MS.	Transient gastrointestinal symptoms (n = 2 on Altilix^®^ and 3 on placebo)
**Curcumin**					
[390]	Randomized, double-blind, placebo-controlled clinical trial	50 patients with MAFLD, 18 y or more	25 patients were assigned to receive placebo or 500 mg of curcumin or placebo/3 times a day/12 weeks	The intake of curcumin was associated with a significant decrease in liver fibrosis (*p* < 0.001) and NF-kB activity (*p* < 0.05). Hepatic steatosis and liver enzymes and TNF-α were significantly reduced in both groups (*p* < 0.05).	NR
[391]	Randomized, double-blind, placebo-controlled study	65 patients allocated to curcumin or placebo	Curcumin and placebo recipient groups using a block randomized design for 8 weeks	There was a significant increase in HDL-c levels in the curcumin group (*p* = 0.01); serum adiponectin increased significantly (*p* < 0.001), and leptin reduced significantly (*p* < 0.001) (decrease in the leptin–adiponectin ratio in the curcumin group).	No AE
[392]	Double-blind parallel design. Iran	54 patients with MAFLD	Phytosomal curcumin (250 mg/day) or placebo/8 weeks	There was a significant reduction in methylation in the promoter regions of the MutL homolog 1 (MLH1) and the MutS homolog 2 (MSH2). A comparison between groups did not indicate significant changes in anthropometric variables, except for BMI. Liver enzymes and 8-OHdG did not change significantly at the end of this study, and neither in the curcumin group nor in the placebo group did they change.	NR
[393]	Prospective, randomized study	45♀ obese women with fatty liver disease	Participants were assigned to resistance training (RT), curcumin supplement, resistance training with curcumin (RTC), and placebo	ALT and AST decreased significantly in the RT and RTC groups (*p* ≤ 0.05) but not in the curcumin and placebo groups (*p* > 0.05). Alkaline phosphatase, total bilirubin, platelet count, and liver structure did not change significantly in all groups. Resistance training alone and with curcumin supplementation could significantly improve liver function, while taking curcumin alone had no significant effect on it.	NR
[394]	Randomized, double-blind, parallel-group, placebo-controlled clinical trial	80 individuals 18 y–70 y (BMI: 25–30 kg/m^2^) and glycemia 100–125 mg/dL	Participants received 2 capsules /day of 800 mg phytosomal curcumin	After 56 days of treatment, the curcumin-treated group showed a significant amelioration in fasting plasma insulin, HOMA index, waist circumference, blood pressure, triglycerides, HDL-c, hepatic transaminases, gamma-GT, hepatic steatosis index, and serum cortisol compared to baseline. Triglycerides, liver transaminases, fatty liver index, and cortisol levels also improved significantly compared to the placebo.	NR
[395]	Double-blind, parallel-group trial	37 obese, non-diabetic individuals	Participants received curcumin or placebo/6 weeks	In comparison to placebo, curcumin showed no significant effects on liver fat content in obese individuals with mild steatosis.	Dyspnea (n = 1)
[396]	Double-blind, randomized trial	80 patients with non-alcoholic simple fatty liver disease	Participants received 500 mg/d curcumin or placebo/24 weeks	There was a significant reduction in the liver fat content, free fatty acid, triglycerides, fasting blood glucose glycated hemoglobin, and insulin	
**Epigallocatechin-gallate**					
[359]	Double-blind, placebo-controlled clinical trial	30 obese subjects were allocated into EGCG-supplemented group or placebo	Participants received 300 mg per day of EGCG for 8 weeks	EGCG significantly reduced systolic and diastolic blood pressure (*p* < 0.05), fasting plasma triglyceride (*p* < 0.05), and serum kisspeptin levels (*p* < 0.05) after the treatment.	Headache (n = 1)
**Puerarin**					
[397]	Randomized, double-blind, placebo-controlled, 2-way crossover trial	217 Chinese ♂, 18–50 y without a history of heart disease	Participants were randomized to receive puerarin (90.2 mg daily) or placebo, followed by a 4-week washout, and then crossed over to the other intervention	No significant modifications were seen in lipid profile, blood pressure, high-sensitive C-reactive protein, liver or renal function after the treatment with puerarin. There was a significant decrease in glycemia.	NR
**Quercetin**					
[398]	Double-blind, placebo-controlled crossover study	93 overweight or obese subjects aged 25–65 y with MS	Participants received 150 mg quercetin a day/six-week treatment periods separated by a five-week washout phase	Participants treated with quercetin showed a significant reduction in systolic blood pressure, but lipid levels, C-reactive protein, and TNF-α were not altered. Quercetin significantly reduced the plasma concentrations of atherogenic oxidized LDL-c.	NR
**Resveratrol**					
[399]	Randomized, double-blind, controlled clinical trial	50 MAFLD patients	Participants received 500 mg resveratrol capsule or a placebo/12 weeks	The group treated with resveratrol had a significantly reduced hepatic steatosis grade, ALT, AST, NFKB, inflammatory cytokines, and serum cytokeratin-18 compared with placebo.	NR
[400]	Double-blind, randomized, placebo-controlled trial	60 participants with MAFLD	Participants received 2150 mg resveratrol capsules or placebo 2 times a day/3 months	The group treated with resveratrol had significantly reduced ALT, AST LDL-c, glycemia, HOMA, total cholesterol TNF-α, cytokeratin 18 fragment, and fibroblast growth factor 21. There was an increase in adiponectin levels.	NR
[401]	Single-center, randomized, double-blind, placebo-controlled study	112 men and women with BMI > 27 kg/m^2^; 18–70 y	Participants received resveratrol 150 mg/day or placebo for 12 weeks	There was a change in liver fat content after treatment as well as in the visceral and subcutaneous abdominal fat mass and a reduction in glycemia, HOMA index, and other cardiovascular risk factors.	NR
[402]	Randomized controlled clinical trial	90 patients with MAFLD (both genera); 20–60 y with BMI 25 to 35 kg/m^2^.	Participants were divided into 3 intervention groups: one that received a low-calorie diet, the resveratrol group received 600 mg pure trans-resveratrol (2 × 300 mg/day), and the placebo group/12 weeks	There was a significant reduction in weight and BMI observed in the resveratrol group compared to the placebo group. No modifications were seen in the lipid profile, ALT, AST, hepatic steatosis grade, serum glycemic parameters, and lipid profiles in the resveratrol group (all *p* > 0.05).	NR
[403]	Randomized, double-blind, placebo-controlled clinical trial	50 patients with MAFLD; 20–60 years	Participants received 600 mg resveratrol/ day or placebo/12 weeks	The use of resveratrol significantly reduced waist circumference, body weight, and BMI when compared to the placebo. No significant modifications were observed in lipid profile (ApoA1, ApoB, and ox-LDL), atherogenic indices, AST, ALT, and γ-GT, and blood pressure.	NR
[404]	Double-blind, randomized controlled trial	76 patients with T2DM	Participants received 1000 mg/day resveratrol or placebo/8 weeks	The supplementation with resveratrol did not produce effects on hepatic steatosis and cardiovascular indices.	NR
**Silymarin**					
[405]	Open preliminary pilot study	85 participants with MAFLD	Patients received silybin + vitamin E + phospholipids—RealSIL	The use of silybin + vitamin E+ phospholipids can improve insulin resistance and the plasma levels of markers of liver fibrosis.	NR
[406]	Preliminary study	59 were affected by primitive MAFLD	Patients received silybin + vitamin E + phospholipids—RealSIL, 4 pieces of silybin (94 mg each piece) /day/6 months followed by more 6 months of follow up	The treated patients showed improved liver enzyme levels, reduced hyperinsulinemia, and an improvement in all liver fibrosis indices.	NR
[407]	Multicenter, phase III, double-blind clinical trial	179 patients with MAFLD	Patients received Realsil (silybin plus phosphatidylcholine) or placebo twice daily/12 months	The treatment with Realsil significantly reduced HOMA, liver enzyme levels, and BMI. There were improvements in fibrogenesis markers.	Diarrhea, dysgeusia, and pruritus
[408]	Randomized clinical pilot study	36 patients with MAFLD	Patients received 2 tablets of silymarin plus vitamin E (Eurosil 85^®^, MEDAS SL)/day/3 months	Associated with lifestyle modifications, silymarin reduced anthropometric parameters, γ-GT levels, and MAFLD index.	NR
[409]	Open-controlled clinical trial	78 patients with MS and liver steatosis	One group received Eurosil 85(^®^) (silymarin + vitamin E), and the other received placebo	The participants who received the silymarin supplement had reduced BMI, abdominal circumference, ultrasound measurement of the right liver lobe, and adipose visceral indices.	NR
[410]	Randomized, double-blind, placebo-controlled trial	99 adults with NASH and MAFLD activity score of 4	Participants received silymarin (700 mg) or placebo 3 times/day/48 weeks	The group treated with silymarin had a significantly reduced fibrosis-AST-to-platelet ratio index, fibrosis-4 score, and MAFLD fibrosis score.	NR
[411]	Double-blind randomized trial	Sedentary men and women with BMI ≤ 34.9 kg/m^2^	Participants were divided into Novel Nutraceutical Supplement without silymarin or with silymarin extract (9%) (4 capsules/day)	There was a reduction in the waist circumference, as well as in the waist-to-height ratio and waist-to-hip ratio AST and ALT, and endocrine hormones cortisol and thyroid-stimulating hormone (TSH) at 90 and 180 days after supplementation with or without silymarin.	NR

Abbreviations: AE: adverse event; ALT: alanine aminotransferase; AST: aspartate aminotransferase; BMI: body mass index; γ-GT: Gamma-Glutamyltransferase; HDL-c: high-density lipoprotein cholesterol; HOMA: Homeostatic Model Assessment; MAFLD: Metabolic-Associated Fatty Liver Disease; MS: metabolic syndrome; NF-kB: nuclear factor kappa B; PBMCs: peripheral blood mononuclear cells; T2DM: type 2 diabetes mellitus.
